# Co-transcriptional RNA cleavage by Drosha homolog Pac1 triggers transcription termination in fission yeast

**DOI:** 10.1093/nar/gkab654

**Published:** 2021-08-05

**Authors:** Carlo Yague-Sanz, Maxime Duval, Marc Larochelle, François Bachand

**Affiliations:** RNA Group, Department of Biochemistry & Functional Genomics, Université de Sherbrooke, Sherbrooke J1E 4K8, Québec, Canada; RNA Group, Department of Biochemistry & Functional Genomics, Université de Sherbrooke, Sherbrooke J1E 4K8, Québec, Canada; RNA Group, Department of Biochemistry & Functional Genomics, Université de Sherbrooke, Sherbrooke J1E 4K8, Québec, Canada; RNA Group, Department of Biochemistry & Functional Genomics, Université de Sherbrooke, Sherbrooke J1E 4K8, Québec, Canada

## Abstract

Transcription termination of protein-coding genes in eukaryotic cells usually relies on a tight coordination between the cleavage and polyadenylation of the pre-mRNA, and 5′-3′ degradation of the downstream nascent transcript. Here we investigated the contribution of the essential fission yeast endonuclease Pac1, a homolog of human Drosha that cleaves hairpin RNA structures, in triggering polyadenylation-independent transcription termination. Using ChIP-sequencing in Pac1-deficient cells, we found that Pac1 triggers transcription termination at snRNA and snoRNA genes as well as at specific protein-coding genes. Notably, we found that Pac1-dependent premature termination occurred at two genes encoding conserved transmembrane transporters whose expression were strongly repressed by Pac1. Analysis by genome editing indicated that a stem-loop structure in the nascent transcript directs Pac1-mediated cleavage and that the regions upstream and downstream of the Pac1 cleavage site in the targeted mRNAs were stabilized by mutation of nuclear 3′-5′ and 5′-3′ exonucleases, respectively. Our findings unveil a premature transcription termination pathway that uncouples co-transcriptional RNA cleavage from polyadenylation, triggering rapid nuclear RNA degradation.

## INTRODUCTION

Ribonucleases (RNases) are found in all domains of life and play important roles in the post-transcriptional control of gene expression through RNA maturation and catabolism. RNases are classified into two main groups depending on whether they cleave within RNA molecules (endoribonucleases) or degrade RNA from its extremities (exoribonucleases). RNase III is an ancient family of endonucleases that specifically target double-stranded (ds) RNA substrates. The first RNase III enzyme was identified in *Escherichia coli* (1), where it plays key roles in the maturation of structured non-coding RNAs (ncRNA), including the primary ribosomal RNA (rRNA) transcript (2), a role that is conserved from prokaryotes to eukaryotes (3).

Additional RNase III substrates have also emerged throughout evolution. For instance, in *Saccharomyces cerevisiae*, the RNase III homolog Rnt1 processes RNA polymerase (RNAP) II-transcribed polycistronic transcripts into individual entities (4,5), participates in the maturation of snoRNAs (6), regulates mRNA stability (7), and can trigger termination of RNAPI and II transcription in a Rat1/torpedo-dependent manner (8,9). The torpedo model for eukaryotic transcription termination relies on nascent transcript cleavage that creates an entry point for a 5′-3′ exoribonuclease (Rat1 in budding yeast), which catches up with the RNAP and ultimately promotes transcriptional termination (10–12). For protein-coding genes, torpedo termination normally relies on nascent transcript cleavage by the cleavage and polyadenylation factor (CPF) complex, which couples termination with pre-mRNA polyadenylation. However, when Rnt1 substitutes for canonical CPF cleavage, polyadenylation-independent termination can occur, which primarily acts as a fail-safe mechanism of transcription termination for RNAPII-transcribed protein-coding genes (8,9), but constitutes the main RNAPI termination pathway at rRNA genes (13).

In humans, there are two RNase III homologs, Drosha and hDicer, that are best known for their role in microRNA (miRNA) biogenesis. miRNAs are short (about 22-nt long) single-stranded RNAs derived from endogenous hairpin dsRNA precursors that are matured by Drosha cleavage activity (14). Functionally, miRNAs are known to regulate cell proliferation and differentiation through RNA interference (RNAi), and are considered as potential targets for human cancer diagnosis, prognosis, and treatment (15). In addition, Drosha has been shown to cleave non-miRNA targets such as mRNAs, influencing transcript stability and/or transcription termination (16,17). These emerging roles of Drosha remain poorly understood, however.

In contrast to budding yeast, the fission yeast *Schizosaccharomyces pombe* also possesses two RNAse III family members: Pac1 and spDicer. While the functions of spDicer are well described, the essential Pac1 protein, homolog to both *S. cerevisiae* Rnt1 and human Drosha, remains poorly characterized. The *pac1* gene was initially isolated as a high copy suppressor of the untimely meiosis phenotype that occurs in a *S. pombe pat1* mutant, and may therefore contribute to fission yeast sexual differentiation, although the underlying mechanism remains unknown (18,19). Functionally, Pac1 cleavage activity is targeted on dsRNA stem-loops of at least 20-nt (20), but tolerates bulges in the stem and extensive variation in the loop length and sequence (21). A *pac1* mutation causes defects in snRNA processing (22,23) and in the 3′ end formation and transcriptional termination of selected snRNAs and snoRNAs (24,25). As yet, however, our current understanding of Pac1 function and substrate specificity is limited to only three substrates. Specifically, a single endogenous substrate has been reported for Pac1, which is the rDNA 3′ external transcribed spacer (3′ETS) (21), whereas the other two Pac1 substrates described previously (the U2 snRNA and U3 snoRNA) were characterized using plasmid-borne expression systems (24,25).

Here, we investigated the endogenous co-transcriptional targets of Pac1 using functional genomic approaches. We found that Pac1 triggers transcription termination at snRNA and snoRNA genes as well as at specific protein-coding genes. Notably, Pac1 activity imposed premature transcription termination and concomitant RNA decay for two genes encoding transmembrane transporters: *mfs2* and *SPBC530.02*. Mutation of a predicted stem-loop structure within *mfs2* by genome editing confirmed that the stem-loop structure in the nascent transcript is responsible for Pac1-dependent repression. Intriguingly, *mfs2* transcriptional induction upon stress occurred independently of the constitutive repression activity of Pac1. Our findings unveil a premature transcription termination pathway that uncouples co-transcriptional RNA cleavage from polyadenylation, triggering rapid RNA degradation in the nucleus.

## MATERIALS AND METHODS

### Yeast strains and media

Unless stated otherwise, cells were grown at 30°C in Edinburgh minimal media (EMM) supplemented with adenine, uracil, histidine, and leucine. Cells were collected at OD600nm of ∼0.5–0.7. A list of all *S. pombe* strains used in this study is provided in Supplementary Table S3. Conditional strains in which the *nmt41* or *nmt81* promoters control the expression of genes of interests were repressed by a 12–15 h treatment with 60 μM of thiamine as previously described (28). Nuclear proteins fused to the anchor-away system (26) were relocalized in the cytoplasm by a two-hours rapamycin treatment at a final concentration of 2.5 μg/ml. Gene disruptions and C-terminal tagging of proteins were performed by PCR-mediated gene targeting (58) using lithium acetate method. Short-homology CRISPR/Cas9-mediated mutagenesis (59) was used to generate the *mfs2 stemdead* and *pac1-ts* mutants.

### Microscopy

Pac1-FRB-GFP localization was detected by using fluorescence microscopy as previously described (28). Briefly, liquid cultures were grown in EMM to early log phase (OD600nm 0.3) then rapamycin or an equal volume of DMSO was added to a final concentration of 2.5 μg/ml. After two hours incubation, nuclei were stained using Hoechst 33342 for 15 min (0.2 mg/ml) and live cells were mounted on 1.2% agarose patches. GFP-tagged proteins and nuclei were detected at 470 nm and 365 nm, respectively, using a Colibri system (Carl Zeiss Canada, Toronto, ON, Canada) on a Zeiss Axio Observer Z1 inverted microscope with a ×60/1.4 oil objective. Data were analyzed using the ZEN black software (Carl Zeiss Canada).

### RNA preparation and analyses

Total RNA was extracted using the hot-acid phenol method, as previously described (60). RT-qPCR analyses were performed as previously described (61). Briefly, 1 μg of total RNA was treated with 1 unit of RNase-free DNase RQ1 (Promega, M6101) for 30 min at 37°C and inactivated with 1 μl of 25 mM EDTA for 10 min at 65°C. Reverse transcription reactions were in a volume of 20 μl using random hexamers and 1 unit of Omniscript RT (Qiagen) for 60 min at 42°C and inactivated for 20 min at 65°C. qPCR reactions were performed in triplicates on a LightCycler 96 system (Roche) in a final volume of 15 μl using 6 μl from a 1:100 dilution of each cDNA, 0.15 μM of forward and reverse primers, and 7.5 μl of the 2X PerfeCTa SYBR Green Supermix from Quantabio. Analysis of gene expression changes were calculated relative to the appropriate control *S. pombe* strain and were measured with the ΔΔCT method using the gene *nda2* as internal reference. The oligonucleotides used in the qPCR experiments are listed in Supplementary Table S4.

rRNA-depleted RNA-seq libraries were prepared following manufacturer's instructions using either the Zymo-Seq RiboFree Total RNA Library Kit (for the *pac1-ts* RNA-seq experiment) or Illumina Truseq Stranded Total RNA-sequencing after ribodeletion with the RiboZero Yeast ribodepletion (Epicentre). RNA-seq libraries were sequenced in paired-end (2 × 50nt) using Illumina technologies (MiSeq or NovaSeq) at Genome Québec.

For northern blot analysis, 8 μg of total RNA were separated on 7% acrylamide gels containing 8% urea. After transfer on a nitrocellulose membrane (Amersham Hybond™), mature and precursors snoRNA were detected using DNA probes radiolabeled with ATP[γ-^32^P] using T4 polynucleotide kinase phosphorylation (NEB), following manufacturer's instruction. Probes were hybridized overnight to the membrane at 42°C.

### *In vitro* cleavage assays

Recombinant Pac1-6xhis (62) was expressed in *E. coli* grown in LB medium at 37°C for 3 hours after IPTG (1 μM final concentration) induction. After centrifugation, cell pellets were resuspended in extraction buffer (25% glycerol, 1 M NaCl, 30 mM tris pH 8, 10 mM imidazole, 1x PLAAC protease inhibitor cocktail) and treated with lysozyme at 1 mg/ml during 30 minutes at 4°C. Six sonication cycles of 10 s at 20% amplitude completed the lysis process. After centrifugation, recombinant Pac1 was purified from cell extracts using Ni-NTA agarose beads (Qiagen) following manufacturer's instructions. Briefly, 50 μl of washed beads were added to a cell extract corresponding to 50 mL of initial bacterial culture and were incubated 60 minutes at 4°C on a rotating wheel. The beads were then washed five times with extraction buffer, then two more times with Pac1 reaction buffer (30 mM CHES pH 8.5, 1 mM DTT, 5 mM MgCl_2_, 0.1 mg/ml BSA). Digestion of Pac1 substrates were directly performed on beads (no elution).

Radiolabeled Pac1 substrates were transcribed by T7 RNA polymerase (NEB) from a DNA template generated by PCR in presence of UTP[α-^32^P] according to the manufacturer's instructions. Following column purification (Zymoresearch RNA clean & concentrator™), the transcription product was heated at 85°C for 5 min then annealed at room temperature for 30 min. The annealed RNA was then added to the purified Pac1 and incubated at 30°C for the indicated times. The cleavage reactions were stopped by the addition of 2× formamide RNA loading, followed by migration on a 8% polyacrylamide gel containing 8M urea.

### Chromatin immunoprecipation (ChIP)

ChIP-qPCR and ChIP-seq experiments were performed as described previously (61). Briefly, 50 mL of OD600nm = 0.6–0.7 cultures were incubated for 20 min at room temperature with 1% formaldehyde. After quenching the reaction with glycine, cells were washed with cold Tris-buffered saline (20 mM Tris–HCl pH 7.5, 150 mM NaCl) and snap-freezed. Frozen pellets were thawed and resuspended in 500 μL of lysis buffer (50 mM HEPES–KOH at pH 7.5, 140 mM NaCl, 1 mM EDTA at pH 8.0, 1% Triton X-100, 0.1% Na-deoxycholate) containing protease inhibitors, disrupted using a FastPrep instrument, and sonicated 12 times for 10 sec at 20% intensity using a Branson digital sonifier. Sonicated chromatin was incubated overnight at 4°C with 50 μl of Pan Mouse IgG Dynabeads (Life Technologies, 11041) only for Pac1-TAP immunoprecipitation, or with Pan mouse IgG beads coupled with 2 μg of 8WG16 antibody for RNAPII immunoprecipitation. After the beads were washed twice with 1 mL of lysis buffer, twice with 1 mL of lysis buffer plus 500 mM NaCl, twice with 1 mL of wash buffer (10 mM Tris–HCl at pH 8.0, 250 mM LiCl, 0.5% NP-40, 0.5% sodium deoxycholate and 1 mM EDTA), and once with 1 mL of Tris-EDTA (TE; 10 mM Tris-HCl at pH 8.0, 1 mM EDTA), the co-immunoprecipitated chromatin was eluted by incubating the beads 15 minutes in elution buffer (50 mM Tris–HCl at pH 8.0, 10 mM EDTA, 1% SDS) at 65°C. After overnight incubation at 65°C for reverse-crosslinking, treatment with proteinase K and DNA extraction by phenol-chloroform, the samples were treated with RNaseA and purified on PCR purification column (Qiagen). For ChIP-qPCR, DNA from the inputs and immunoprecipitated fractions were analyzed on a LightCycler 96 Instrument system (Roche) using perfecta SYBR supermix (QuantaBio). The oligonucleotides used in the qPCR experiments are listed in Supplementary Table S4. Protein occupancy was then calculated using the percent input method (61). ChIP-sequencing libraries were prepared using the SPARK DNA Sample Prep Kit Illumina Platform (Quantabio) according to the manufacturer's instructions and sequenced in single-end (50nt) using Illumina technologies (MiSeq or NovaSeq) at Genome Québec.

### RNA-seq analysis

The RNA-seq datasets used in this study are listed in Supplementary Table S5. Raw paired reads were trimmed using trimmomatic (63) against the appropriate adapter sequences with options ‘PE ILLUMINACLIP:$adapters:2:30:10 LEADING:3 TRAILING:3 SLIDINGWINDOW:4:15 MINLEN:36’. Trimmed reads were then mapped on *S. pombe* genome (version ASM294v2.26) using HISAT2 (64) with option ‘–RNA-strandness RF –min-intronlen 20 –max-intronlen 3000 –no-mixed –no-discordant’. Then, mapped reads were summarized at the gene level using featureCounts (65) with options ‘-s 2 -p -P -d 0 -D 5000 -C’. Gene-level read count matrix was then inputed into DESeq2 (66) that uses a median of ratio method to compute size factors (normalization factor). Based on these size factors, we used deepTools (67) to create strand-specific normalized coverage files (.bigwig) with options ‘bamCoverage –samFlagExclude 256 –maxFragmentLength –scaleFactor $size_factor –filterRNAstrand [forward,reverse] -bs 1 -of bigwig’. Replicate bigwig files were then merged with deepTools ‘bigWigCompare -bs 1 –operation mean’ and visualized within the Integrative Genomic Viewer (68). Differential expression analysis was carried out in DEseq2 using a generalized linear model of the form ‘expression ∼ strain + temperature + temperature:strain’ to accounts for the interaction between the thermosensitive mutation and the temperature in the case of the *pac1-ts* dataset, and of the form ‘expression ∼ strain’ for the other datasets (Supplementary Table S1).

### ChIP-seq analysis

The ChIP-seq datasets used in this study are listed in Supplementary Table S5. Raw reads were trimmed using trimmomatic (63) against the appropriate adapter sequences with options ‘SE ILLUMINACLIP:$adapters:2:30:10 LEADING:3 TRAILING:3 SLIDINGWINDOW:4:15 MINLEN:36’. Trimmed reads were then mapped on *S. pombe* genome (version ASM294v2.26) using HISAT2 (64) with option ‘–no-splice-alignment’. For input-controlled peak calling of the Pac1-TAP ChIP experiments, MACS2 (69) was used on individual replicates with options ‘-f BAM -t IP.bam -c INPUT.bam -g 12000000 -B -q 0.01 -m 1 10 –fe-cutoff 2 –keep-dup all’. As of note, removing the ‘–keep-dup all’ option did not significantly alter the peak calling results with the exception of the loss of the 3′ETS Pac1 peak because of saturating read coverage arising from the regions harboring the multi-copy rDNA repeats. Individual narrowPeak files (MACS2 output) were converted into bed format and combined using MSPC (Multiple Sample Peak Calling) (70) using parameters ‘-r bio -s 1E–8 -w 1E–4 -c 2’ to obtain the final list of Pac1-associated loci presented in Supplementary Table S2. For visualization, MACS2 pileup.bdg files were converted into bigwig files, the replicates merged, and loaded into the Integrative Genomic Viewer (68). The 8WG16 ChIP dataset (in the control and Pac1-AA strain) was processed similarly, with the exception that the genome coverage tracks were normalized based on a scaling factor computed from the median of ratio of the read coverage of consecutive 10Kb genomic bins obtained using deepTools (67) mutiBigwigSummary bins, allowing for direct comparison of the mutant and control profiles.

### RNA structure prediction and conservation.

Secondary RNA structure were predicted using the RNAfold web server (71) and visualized in vaRNA (72). *mfs2* homologs were identified through Fungal Compara, a tool from *Ensembl Fungi* where fungal gene families are generated based on the best reciprocal familiarity relationship (73).

### Data availability statement

Raw reads files and processed files for the RNA-seq (bigwig and read count matrix) and ChIP-seq (bigwig) data generated in this study are available on GEO under accession numbers GSE167041 and GSE167040, respectively.

## RESULTS

### Pac1 associates with chromatin and promotes transcription termination of snRNA and snoRNA genes

**Figure 1.**
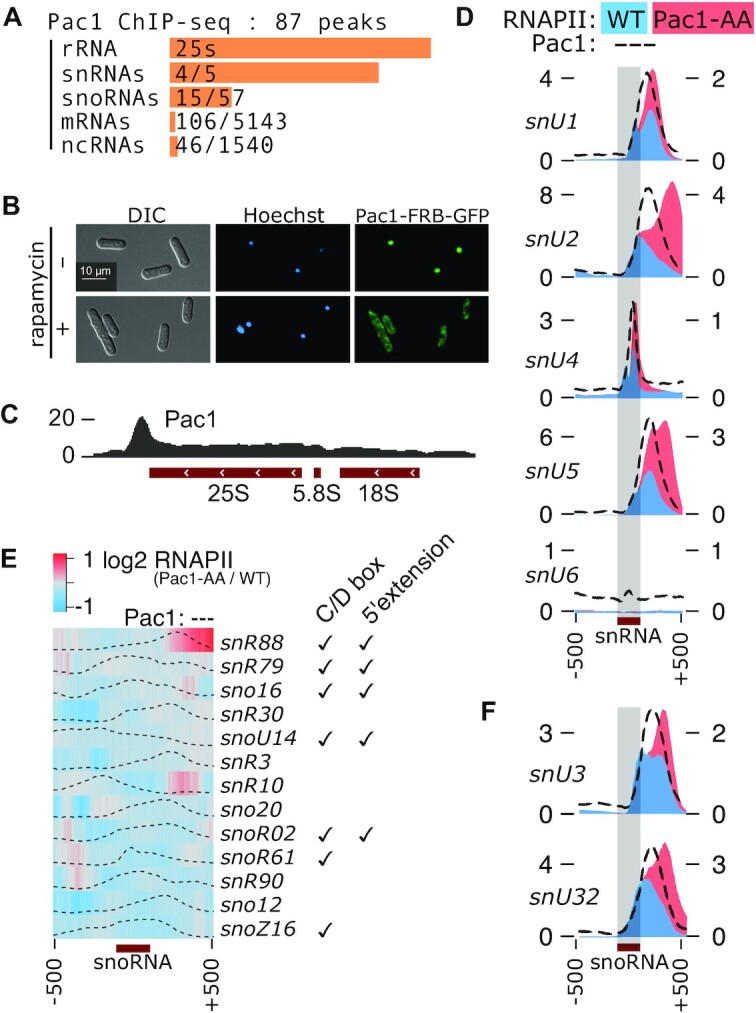
Pac1 associates with chromatin and promotes transcription termination of snRNA and snoRNA genes. (**A**) Distribution by type of the 172 genes overlapping the 87 Pac1-associated loci identified by ChIP-sequencing (Supplementary Table S1). The orange bars are proportional to the percentage of genes bound by Pac1 for each class of genes. (**B**) Representative images of the Pac1 anchor away strain (Pac1-FRB-GFP) natural nuclear localization (top panel) and relocalization to the cytoplasm 2h after rapamycin treatment (bottom panel). (**C**) Pac1-TAP ChIP-seq read coverage on one representative rDNA repeat, transcribed from right to left. The coverage is expressed in thousands reads mapped and averaged over two biological replicates. (**D**, **F**) Normalized ChIP-seq read coverage over snRNA (D) and selected snoRNA (F) genes for RNAPII (8WG16 antibody) in the Pac1 anchor-away (Pac1-AA, red) or control strains (blue) 2h after rapamycin treatment (right axis) and for Pac1-TAP (left axis, dotted black line). The coverage is expressed in thousands reads mapped and averaged over two biological replicates. (**E**) Heatmap of the log2 ratio between the normalized RNAPII ChIP-seq read coverage in the Pac1 anchor-away strain versus a control strain 2h after rapamycin treatment over Pac1-bound snoRNAs genes (500-nt upstream and downstream of snoRNA annotation). The Pac1-TAP ChIP signal distribution (min-max scaled to fit the window) is represented by dotted black lines. 5′ extension refers to the accumulation of 5′-extended snoRNA precursors upon Pac1 nuclear exclusion (see Supplementary Figure S3).

To identify transcripts co-transcriptionally cleaved by Pac1—a process that presumably occurs in close proximity to the chromatin template—we performed Pac1 ChIP-sequencing using a strain expressing a functional TAP-tagged version of Pac1 from its endogenous chromosomal locus. Peak calling on the resulting reads revealed that Pac1 is significantly associated with almost 200 genomic loci covering rRNA, snRNA, snoRNA, mRNA and lncRNA genes (Figure 1A & Supplementary Table S1). Among these loci, we recovered the genes encoding all three previously identified Pac1 targets: the rRNA 3′ External Transcribed Sequence (ETS) (20), snU2 (24), and snU3 (25) (Supplementary Table S1), confirming that ChIP-seq assays are appropriate to identify transcripts targeted by Pac1.

To assess whether the essential Pac1 protein is functionally important for transcription of the identified putative targets, we used a conditional anchor-away (26) Pac1 strain (Pac1-AA) that allowed rapid rapamycin-dependent nuclear exclusion of Pac1 (Figure 1B). Long-term rapamycin treatment did not support cellular growth of the Pac1-AA strain (Supplementary Figure S1A). We therefore limited the rapamycin treatments to 2 h in all of our experiments to avoid indirect effects caused by cellular mortality. Using our conditional *pac1* mutant, we questioned the fate of RNAPI and II occupancy after Pac1 nuclear exclusion using both ChIP-qPCR and ChIP-seq assays. In the case of RNAPI, Pac1 robustly bound the rDNA repeats in the 3′ETS region downstream of the 25s rDNA (Figure 1C). This is consistent with previous findings showing that the 3′ETS stem-loop is a Pac1 target *in vivo* and *in vitro* (20,21). Because of this association, Pac1 activity at the 3′ETS was thought to be important for termination of RNAPI transcription. Surprisingly, nuclear exclusion of Pac1 did not significantly affect the distribution of the RNAPI subunit Rpa2 at the rDNA termination region (Supplementary Figure S1B), suggesting either that Pac1 activity is not required for RNAPI termination or that fail-safe mechanisms allow efficient RNAPI termination in the absence of Pac1 (see Discussion).

Besides the rDNA, the most enriched class of Pac1-bound genes were snRNA genes. Indeed, *snU1*, *snU2*, *snU4*, and *snU5* were strongly associated with Pac1, whereas the *snU6* gene (transcribed by RNAPIII) was not (Figure 1D, black dotted lines). Strikingly, RNAPII accumulation occurred downstream of the four Pac1-bound snRNA genes upon Pac1 nuclear exclusion (Figure 1D, compare red and blue profiles). Previous studies on Pac1 and its orthologs have identified minimal *in vitro* requirements for Pac1 substrates, including a dsRNA helix of at least 20-nt with potential internal bulges (20). We therefore analyzed the sequence downstream of mature snRNA units for the presence of RNA secondary structures that can represent putative Pac1 substrates. Notably, for all snRNA genes transcribed by RNAPII (*snU1*, *snU2*, *snU4*, and *snU5*), stable hairpin structures with stems of 23–32 nucleotides ending with small loops of 3–5 nucleotides were predicted directly downstream of the annotated mature snRNA units (Supplementary Figure S2A). Together with the observed RNAPII termination defects, the predicted RNA hairpin structures support that Pac1 promotes transcription termination of endogenous snRNA genes in fission yeast.

Pac1 also associated with a total of 15 snoRNA genes, but only snoRNA genes expressed from independent transcription units (*i.e*., no intronic snoRNAs nor polycistronic snoRNA clusters) (Supplementary Figure S2B). Although Pac1 was previously shown to function in transcription termination of the *snU32* snoRNA gene expressed from a plasmid-borne transgene (25), our data revealed that Pac1 nuclear exclusion did not result in widespread readthrough of RNAPII transcription at snoRNA genes (Figure 1E), with the notable exceptions of *snU3*, *snU32* and *snR88* (Figure 1E and F). Accordingly, stable RNA hairpin structures that can function as Pac1 substrates are also predicted downstream of the annotated 3′ end of *snU3* and *snU32* snoRNAs (Supplementary Figure S2A). While both C/D and H/ACA box snoRNA genes were bound by Pac1 (Supplementary Figure S2B), a difference between the two groups is that Pac1 nuclear exclusion specifically led to the accumulation of 5′-extended precursors of Pac1-bound C/D box snoRNAs (Supplementary Figure S3A). This accumulation was confirmed by Northern blot assays on three C/D box snoRNAs (*sno16*, *snoU14* and *snr79*), whereas a control H/ACA box snoRNA (*sno12*) was unaffected by Pac1 nuclear exclusion (Supplementary Figure S3B). Furthermore, stable hairpin structures were predicted either directly upstream or up to 50 nucleotides upstream of the annotated mature 5′ end of the C/D box snoRNAs that showed accumulation of 5′-extended precursors by Northern blotting (Supplementary Figure S3C). These observations are reminiscent of the role of the *S. cerevisiae* Pac1 ortholog Rnt1 in the co-transcriptional 5′-end processing and maturation of C/D box snoRNAs (6).

Collectively, our data reveal that Pac1 is recruited to an extensive set of coding and non-coding RNA genes with a key role in transcription termination at snRNA and selected snoRNA genes.

### Pac1 triggers premature transcription termination at protein-coding genes

**Figure 2.**
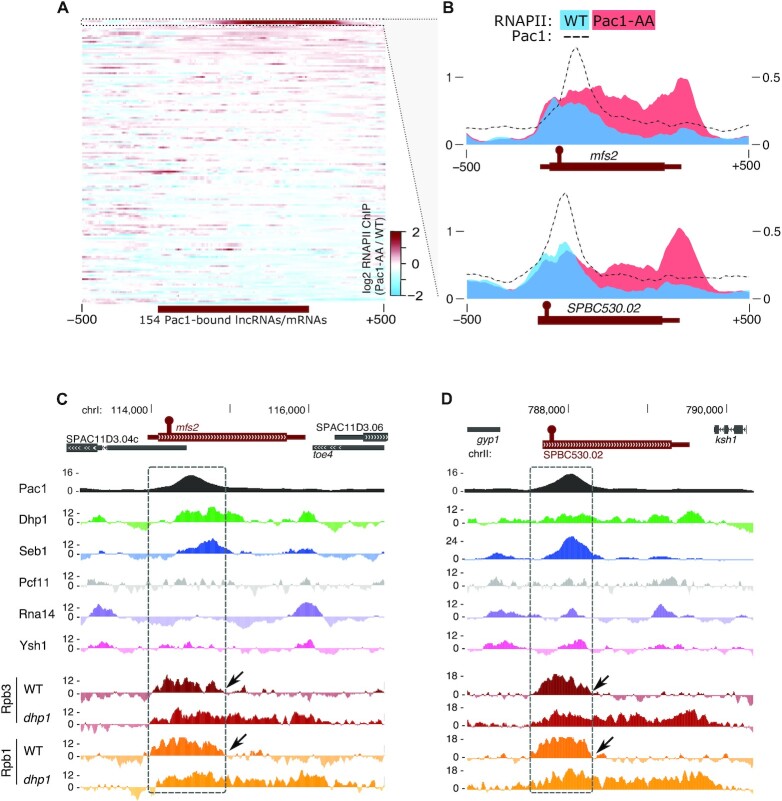
Pac1 triggers premature transcription termination at protein-coding genes. (**A**) Heatmap of the log2 ratio between the normalized RNAPII ChIP-seq read coverage over Pac1-bound mRNA/lncRNA genes in the Pac1 anchor-away strain versus a control strain 2h after rapamycin treatment. (**B**) Normalized RNAPII ChIP-seq read coverage (right axis) over the *mfs2* and *SPBC530.02* genes in the Pac1 anchor-away (Pac1-AA, in red) or control strain (blue) 2h after rapamycin treatment and for Pac1-TAP (left axis, dotted black line). The coverage is expressed in thousands reads mapped and averaged over two biological replicates. (**C**, **D**) Pac1 ChIP-seq coverage (expressed in thousands reads mapped and averaged over two biological replicates) and normalized ChIP-seq read coverage for the indicated termination factors and for RNAPII (Rpb1 and Rpb3 subunits) in wild-type cells and after Dhp1 depletion in a strain where *dhp1* is under the control of the thiamine-sensitive *nmt81* promoter over the *mfs2* (C) and *SPBC530.02* (D) genes.

Having validated our approach to identify transcripts cleaved by Pac1 on expected Pac1 targets, we next sought to explore the relevance of Pac1 association with the 150 putative new targets in the protein-coding and lncRNA genes categories (Figure 1A and Supplementary Table S1), in particular regarding RNAPII termination. While most Pac1-bound mRNA and lncRNA genes showed only minor differences in RNAPII occupancy after Pac1 nuclear exclusion, important changes were observed for two protein-coding genes: *mfs2* and *SPBC530.02* (Figure 2A and B). *mfs2* and *SPBC530.02* are paralogous genes and share 66% identity across their full-length ORF. They are predicted to encode for proteins composed of eleven transmembrane domains typical of the major facilitator superfamily (*mfs*) of transporters (27).

The RNAPII profiles at *mfs2* and *SPBC530.02* were similarly affected by Pac1 nuclear exclusion: in the control strain, we observed a sharp decline in RNAPII occupancy inside the gene body, directly downstream of the Pac1-bound region located in the first half of the genes (Figure 2B, blue profile). In contrast, Pac1 nuclear exclusion resulted in extended RNAPII occupancy throughout the entire ORFs (Figure 2B, red profile). Such differences in RNAPII profiles are suggestive of Pac1-dependent premature termination. Accordingly, the termination-associated Rpb1 CTD marks, Ser2 and Tyr1 phosphorylation (28), accumulated specifically at the putative premature transcription termination sites, whereas the CTD Ser5 and Ser7 phosphorylation marks were distributed throughout the 5′ half of the gene (Supplementary Figure S4A). The sites of premature termination were also bound by the termination factor Seb1 (Figure 2C-D), although Seb1 might not be critical for premature termination at *mfs2* and *SPBC530.02* genes as the RNAPII distribution remained unaffected after Seb1 depletion (Supplementary Figure S4B).

Transcription termination by the torpedo model typically occurs following CPF recruitment to the nascent transcript, which triggers CPF-dependent cleavage followed by RNAPII disengagement from chromatin by the 5′-3′ exonuclease complex (10,29). In the case of the putative premature termination occurring at *mfs2* and *SPBC530.02* genes, we hypothesized that Pac1-dependent cleavage substitutes for canonical CPF cleavage to trigger torpedo-dependent termination. In support for this model, we used previously published ChIP-seq datasets (28,30) to show that CPF components Ysh1, Rna14, and Pcf11 were poorly associated with the premature termination sites of *mfs2* and *SPBC530.02*, in contrast to the torpedo nuclease Dhp1 (Figure 2C and D). In addition, RNAPII accumulated in the second half of the *mfs2* and *SPBC530.02* genes in Dhp1-deficient cells (Figure 2C and D), a result similar to what was observed upon Pac1 cytoplasmic sequestration (Figure 2B). In contrast, nuclear exclusion of the Ysh1 CPF endonuclease did not increase RNAPII occupancy across the coding region (Supplementary Figure S4B). Taken together, our data indicate that Pac1 promotes premature transcription termination at specific protein-coding genes.

### Pac1-dependent co-transcriptional cleavage restricts gene expression

**Figure 3.**
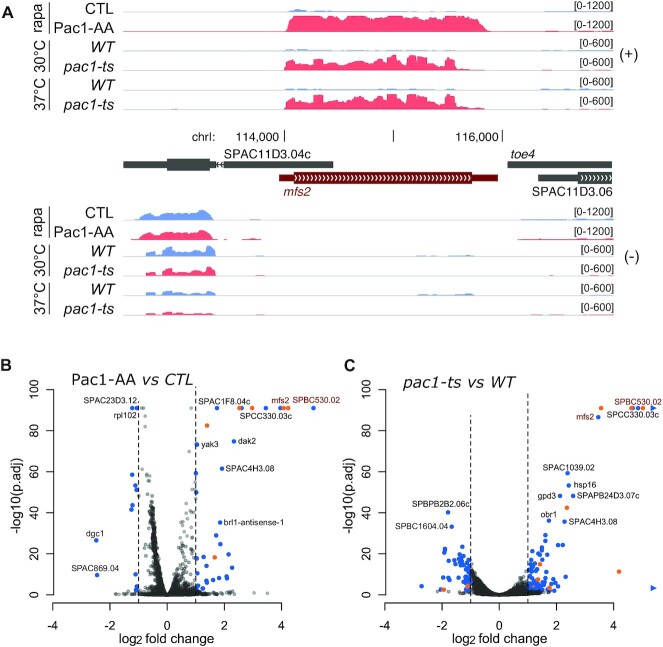
Pac1-dependent co-transcriptional cleavage prevents gene expression. (**A**) Normalized RNA-seq read coverage averaged over two replicates centered on the *mfs2* gene in control (CTL) strains or upon rapamycin-dependent Pac1 nuclear depletion (Pac1-AA) or Pac1 inactivation in a thermosensitive mutant (*pac1-ts*) grown at the semi-restrictive temperature of 30°C or shifted two hours at the restrictive temperature of 37°C. The expression of the annotated long 5′UTR of *SPAC11D3.04c* overlapping *mfs2* on its opposite strand was not detected in our assays. (**B**, **C**) Volcano plot of the log2 fold-change and statistical significance (-log10 of the false discovery rate (FDR)-adjusted *p-value*) of the Pac1-AA strain (B) and the *pac1-ts* mutant (C). Significantly differentially-expressed genes (absolute log2 fold-change >1 and –log_10_ of FDR > 2) are highlighted in blue for protein-coding genes and long non-coding RNA genes, and in orange for snRNA and snoRNA genes. To ease viewing, genes with values beyond axes limits are represented by arrowheads.

Our results support a model in which co-transcriptional cleavage of *mfs2* and *SPBC530.02* nascent transcripts by Pac1 creates an entry point for the 5′-3′ exonuclease Dhp1 complex, leading to premature RNAPII termination. This model predicts that *mfs2* and *SPBC530.02* expression should be repressed in wild-type cells, as such a CPF-independent endonucleolytic cleavage is not expected to support 3′ end polyadenylation of the nascent transcript. To explore this possibility, we performed RNA-seq using the conditional Pac1-anchor away strain. As predicted, we found that *mfs2* and *SPBC530.02* were mostly repressed in control cells (∼10 FPKM in our RNA-seq experiments, <1 RNA copy by cell (31)), but strongly upregulated after Pac1 nuclear exclusion (Figure 3A and Supplementary Figure S5A).

Although the Pac1 anchor-away strain allowed for rapid conditional nuclear exclusion (Figure 1B), it does not preclude the relocalized protein from having an activity in the cytoplasm. To confirm that increased expression of *mfs2* and *SPBC530.02* is caused by the loss of Pac1 activity in the nucleus and not as a consequence of Pac1 activity in the cytoplasm, we took advantage of a previously described thermosensitive *pac1* allele called *snm1-1* (22). As the original *snm1-1* mutant strain is no longer available, we recreated the G1024A single nucleotide mutation of *snm1-1* using CRISPR/Cas9 genome editing, which substitutes the alanine at position 342 for a threonine in the dsRNA-binding domain of Pac1. This strain, that we now refer to as *pac1-ts*, shows the expected thermosensitive phenotype, with a mild growth defect at 26°C and 30°C, and loss of growth at 37°C (Supplementary Figure S5B). RNA-seq analysis of two independent *pac1-ts* clones and a wild-type parental control strain revealed an increase of *mfs2* and *SPBC530.02* mRNA expression in the *pac1-ts* mutant, similarly as in the Pac1 anchor-away strain, confirming that loss of Pac1 nuclear function is the cause of the observed gene expression changes (Figure 3A and Supplementary Figure S5A). Intriguingly, the *pac1-ts* mutation had the same effect on *mfs2* and *SPBC530.02* expression at the semi-restrictive temperature of 30°C than after a 2 h shift at the lethal temperature of 37°C.

A global analysis of gene expression changes in the Pac1-AA strain and the *pac1-ts* mutant showed that, in general, nuclear exclusion of Pac1 in the Pac1-AA strain (Figure 3B) had less impact on gene expression than in the *pac1-ts* mutant, regardless of temperatures (Figure 3C). In total, 17 genes were downregulated in both the Pac1-AA and *pac1-ts* strains (Supplementary Table S2), including the Pac1-bound RNA component of the RNase P complex, *rrk1*. We also found 49 upregulated genes common to both *pac1* mutants (Supplementary Table S2), including 5 Pac1-bound C/D box snoRNAs whose 5′-extended precursors accumulated upon Pac1 nuclear exclusion (Supplementary Figure S3), 15 lncRNAs (13 of which are antisense lncRNAs), and 28 protein-coding genes. Among them, only *mfs2* and *SPBC530.02* were found co-transcriptionally associated with Pac1, as determined by Pac1-TAP ChIP-seq analysis. In addition, these two paralogs were the most highly overexpressed genes, highlighting the importance and specificity of Pac1-dependent gene repression (Figure 3B-C).

### A stem-loop structure in the nascent *mfs2* transcript is required for Pac1-dependent gene regulation

Our data indicate that Pac1 nuclear activity promotes premature transcription termination of *mfs2* and its paralog, resulting in robust repression of their expression. Specifically, the association of Pac1 with *mfs2* demonstrated by ChIP assays (Figure 2) supports that Pac1 acts directly on nascent transcripts. To clarify the mechanism underlying Pac1 recruitment at *mfs2* and *SPBC530.02*, we aimed to identity *cis*-acting elements in the *mfs2* and *SPBC530.02* transcripts that would trigger Pac1 endonucleolytic activity.

**Figure 4.**
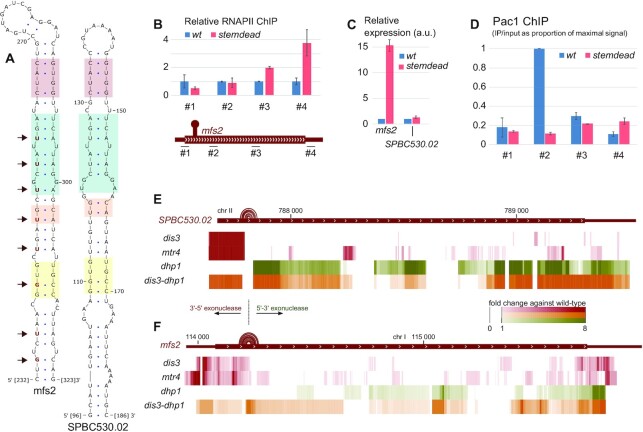
A stem-loop structure in the *mfs2* nascent transcript is a Pac1 substrate. (**A**) Predicted RNA secondary structure at the beginning of *mfs2* and *SPBC530.02* open reading frame. The nucleotides mutated in the *mfs2 stemdead* mutant are shown in red and indicated by arrows. Colored blocks highlight regions of high structural and sequence similarity between the two paralog genes. The nucleotide positions are numbered relative to the beginning of the genes. (**B**) *Top*, ChIP-qPCR analysis of RNAPII relative occupancy (8WG16 antibody) across the *mfs2* gene in wild-type and *mfs2 stemdead* mutant. *n* = 3 biological replicates. *Bottom*, schematic of the *mfs2* gene with bars showing the positions of PCR products analyzed. (**C**) Relative gene expression analysis determined by RT-qPCR in the *mfs2 stemdead* mutant normalized to the *nda2* housekeeping gene and expressed relative to the wild-type control strain. The expression of full-length *mfs2* was measured using amplicon #3 (as in figure 4.B). a.u.: arbitrary units. *n* = 3 biological replicates. (**D**) ChIP-qPCR analysis of Pac1-TAP occupancy at the *mfs2* gene in wild-type and *mfs2 stemdead* mutant. The amplicon numbers refer to the same regions as in (B). *n* = 3 biological replicates. (**E**, **F**) Heatmap of the fold-change of the normalized RNA-seq coverage upon thiamine-dependent depletion of the 3′-5′ exonuclease Dis3 (in red), of its cofactor Mtr4 (in red), of the 5′-3′ exonuclease Dhp1 (in green), or of the double Dis3/Dhp1 depletion (in orange) relative to their appropriate wild-type strain on the *SPBC530.02* (E) and *mfs2* (F) genes. Hairpin RNA secondary structures are represented by arcs connecting base pairs on top of the gene annotation.

We therefore scanned the *mfs2* sequence for secondary structures and found that a 37-nt stem-loop, conserved in the *SPBC530.02* paralog, can form at the beginning of the ORF in both RNAs (Figure 4A). *In vitro* cleavage assays using recombinant protein revealed that Pac1 can recognize and cleave the stem-loop structure found in the *mfs2* transcript (Supplementary Figure S6A). Next, we used CRISPR/Cas9 to introduce eight conservative point mutations that are predicted to disrupt the RNA secondary structure without affecting *mfs2* coding potential (Supplementary Figure S6B). We called the resulting strain ‘*mfs2 stemdead*’. Notably, the *mfs2 stemdead* mutant recapitulated the derepression of *mfs2* observed in *pac1* mutants: compared to the wild-type, RNAPII levels were increased in the 3′ half of *mfs2* (Figure 4B, see regions 3–4) and *mfs2* expression was increased by 15-fold (Figure 4C). Importantly, Pac1 association with *mfs2* was lost in the *stemdead* mutant (Figure 4D). Taken together, these results indicate that the repressive action of Pac1 on *mfs2* expression is directed by the stem-loop structure in the nascent RNA.

Pac1-mediated endonucleolytic cleavage of nascent RNA transcripts is expected to create entry points for 3′-5′ and 5′-3′ exonuclease complexes. To further support the idea that the identified structures within *mfs2* and *SPBC530.02* are subject to endonucleolytic cleavage by Pac1, we questioned the fate of the transcriptomic regions upstream and downstream of the predicted stem-loop structure. Using RNA-seq data from a *dis3* conditional mutant of the 3′-5′ RNA exosome exonuclease complex as well as from a *mtr4* conditional mutant of the exosome cofactor complex TRAMP (32), we observed stabilization of the region upstream of the predicted stem-loop structures (Figure 4E and F). Conversely, the downstream region was stabilized after depletion of the 5′-3′ exonuclease Dhp1 (Figure 4E and F), and both the upstream and downstream regions were stabilized in a double depletion of Dis3 and Dhp1 (Figure 4E and F). Together, our data disclosed a stem-loop structure in the *mfs2* and *SPBC530.02* transcripts that is responsible for Pac1-dependent premature termination of RNAPII transcription and subsequent nuclear degradation of the targeted transcripts.

### Regulation of *mfs2* expression during oxidative stress is independent of Pac1

Our results indicate that *mfs2* and its paralog are constitutively repressed by Pac1. We therefore wondered whether this repression is subject to regulation. To address this question, we scanned the literature, the Pombase database (27), and publicly available datasets (33,34) for conditions where *mfs2* and/or its paralog are upregulated, reasoning that such gene activation may reflect a relief of Pac1-dependent repression.

**Figure 5.**
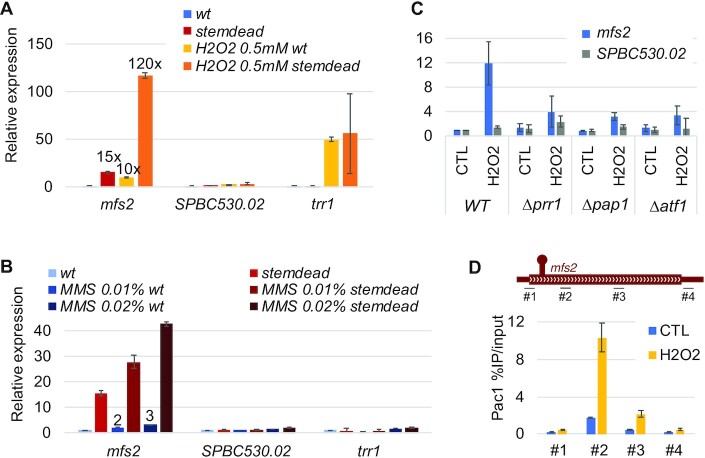
Regulation of *mfs2* expression during oxidative stress is independent of Pac1. (**A**, **B**) Relative gene expression analysis of the indicated genes determined by RT-qPCR in a wild-type strain and the *stemdead* mutant that were treated with H_2_O_2_ (A) or MMS (B). a.u.: arbitrary units. *n* = 3 biological replicates. (**C**) RT-qPCR analysis of *mfs2* and *SPBC530.02* gene expression after 45 min 0.5 mM H_2_O_2_ treatment in the indicated strains. *n* = 3 biological replicates. (**D**) *Top*, Bars *under* the *mfs2* gene show the positions of PCR products used for ChIP analyses. *Bottom*, ChIP-qPCR analysis of Pac1-TAP occupancy on the *mfs2* gene upon 45 min H_2_O_2_ 0.5mM treatment. *n* = 3 biological replicates.

Whereas we have yet to find an environmental condition where *SPBC530.02* is strongly derepressed, *mfs2* is considered as one of the core oxidative response genes (35). Indeed, transcriptomic data from independent studies indicate that *mfs2* is robustly induced upon a variety of oxidative stress conditions (33,34). In our hands, a 45-minute treatment with 0.5 mM hydrogen peroxide (H_2_O_2_)—controlled with the well-described oxidative stress response gene *trr1*—reproducibly induced *mfs2* expression ∼10-fold (Figure 5A). Despite this strong induction, its biological relevance remains unclear as neither the single *mfs2*Δ mutant nor the double *mfs2*Δ *SPBC530.02*Δ mutant showed increased H_2_O_2_ sensitivity compared to wild-type fission yeast (Supplementary Figure S7A). In contrast, we found that deletion of *mfs2* sensitizes cells to treatment with the DNA-damaging agent methyl methanesulfonate (MMS) (Supplementary Figure S5B), while such treatment induces *mfs2* expression in a dose-dependent manner up to 3-fold (Figure 5B). We also noted that the *mfs2 stemdead* mutations did not negatively affect Mfs2 function in MMS resistance (Supplementary Figure S7B), providing evidence that the silent mutations do not affect *mfs2* coding potential.

Next, we reasoned that if *mfs2* induction in oxidative stress is dependent on Pac1, treating the *mfs2 stemdead* mutant with H_2_O_2_ should not further induce *mfs2* expression. However, we observed an almost multiplicative effect between the *stemdead* mutation and the H_2_O_2_ treatment, with a 120-fold induction compared to the wild-type untreated condition (Figure 5A). A similar multiplicative effect was observed when combining MMS treatment with the *stemdead* mutation (Figure 5B). Together, these results indicate that, regardless of the cell growth conditions tested, Pac1 activity is responsible for the repression of about 90–95% of *mfs2* transcripts. This strongly suggests that Pac1 and stress (H_2_O_2_ or MMS treatment) regulate *mfs2* expression through independent mechanisms.

Based on the aforementioned results, we therefore suspected that oxidative stress could stimulate *mfs2* expression independently of Pac1 repression through increased RNAPII initiation. Indeed, two CRE motifs (TGACGT), known to recruit the stress response transcription factors Atf1, Pap1 as well as the Pap1-Prr1 heterodimer (36) are found within 1-kb upstream of the *mfs2* gene. Consistent with stress-induced transcriptional activation, deleting the genes encoding any of those transcription factors (Prr1, Pap1 and Atf1) strongly reduced *mfs2* induction upon H_2_O_2_ treatment (Figure 5C), supporting the view that increased promoter activity is responsible for *mfs2* induction in oxidative stress. We next wondered whether the level of Pac1 association with *mfs2* is affected in oxidative stress, as increased RNAPII initiation from the *mfs2* promoter is expected to increase the amount of Pac1 substrate (i.e. *mfs2* stem-loop). ChIP-qPCR analysis indeed revealed a clear increase in Pac1 association with *mfs2* upon H_2_O_2_ treatment (Figure 5D). We conclude that the induction of *mfs2* in oxidative stress is primarily mediated by a transcriptional activation that surpasses repression by Pac1-dependent premature transcription termination.

## DISCUSSION

In this study, we characterized the roles of the conserved endoribonuclease Pac1 in the regulation of gene expression. Using genomic and transcriptomic approaches, we highlighted transcripts co-transcriptionally targeted by Pac1 and assessed the functional impact of a Pac1 deficiency on gene expression and RNA polymerase transcription.

We show here that Pac1 co-transcriptional cleavage can trigger RNAPII transcription termination at snRNA genes and at a selection of snoRNA and protein-coding genes (Figure 1 and Figure 2A and B). We note, however, that the breadth of termination defects observed upon Pac1 inactivation were different between snRNA/snoRNA and protein-coding genes: the extent of RNAPII readthrough was reduced for snRNA/snoRNA genes (Figure 1D and F) compared to *mfs2*/*SPBC530.02* (Figure 2B). A likely explanation is the redundant role of the mRNA 3′ end processing machinery (CPF) acting as a fail-safe transcription termination pathway directly downstream from where Pac1 is normally involved at snRNA/snoRNA genes. In contrast, the signals for CPF-dependent cleavage would be further downstream for *mfs2* and its paralog (at 3′ end of genes). Accordingly, we have analyzed the distance between the Pac1 cleavage site (stem-loop) and the next poly(A) signal consensus sequence (AAUAAA), and this correlates with the extent of the termination defects observed upon Pac1 inactivation (Supplementary Figure S8). Also consistent with this view is the fact that we have previously shown that mRNA 3′ end processing factors are recruited at the end of ncRNA genes in fission yeast, including snoRNAs and snRNAs (28).

**Figure 6.**
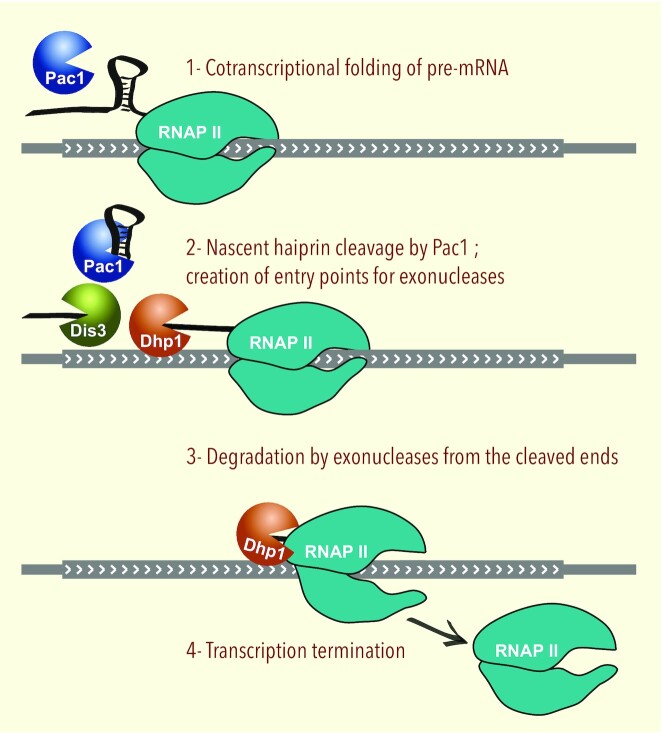
Model of Pac1-mediated gene repression. Endonucleolytic cleavage in the nascent transcript by Pac1 triggers transcription termination of elongating RNAPII by the torpedo model (Dhp1) as well as degradation of the 5′ fragment by the RNA exosome (Dis3).

Pac1-dependent transcription termination resembles normal torpedo termination (10), as Pac1 cleavage creates an entry point for a 5′-3′ exonuclease complex (that includes Dhp1 in *S. pombe*), which elicits RNAPII disengagement from the DNA template. However, as Pac1 recruitment occurs independently from the CPF complex (Figure 2C and D), nascent transcript cleavage is uncoupled from the polyadenylation process, in sharp contrast with canonical CPF-dependent mRNA 3′ end processing (37). Whereas highly structured RNAs such as snoRNAs or snRNAs are intrinsically stable transcripts, pre-mRNAs usually rely on polyadenylation and poly(A)-binding proteins to achieve stability (38). Accordingly, we have shown that mRNAs co-transcriptionally cleaved by Pac1 were subsequently degraded by both 3′-5′ and 5′-3′ exonucleases, and that their expression was robustly increased by a Pac1 deficiency either through Pac1 nuclear exclusion, a Pac1 loss-of-function mutation, or by disrupting an RNA stem-loop structure targeted by Pac1 (Figures 3 and 4). In addition, we found that Pac1-mediated repression is reinforced by the fact that Pac1 cleavage occurs at the beginning of the nascent pre-mRNA and thereby elicits premature termination of RNAPII transcription. Our study thus goes beyond previous findings for RNAse III homologs in budding yeast (8,9) and mammals (17,39) by reporting a mechanism of gene repression that depends on premature transcription termination whereby Pac1-dependent endonucleolytic cleavage couples RNAPII termination with pre-mRNA degradation (Figure 6). Furthermore, the mechanism of Pac1-mediated gene repression described in this study differs from a mode of gene silencing in fission yeast that depends on CPF recruitment in gene bodies to specify targeting by heterochromatin factors (40,41).

Although the mechanism of Pac1-mediated gene repression appears both highly specific and efficient, its biological relevance remains elusive. An attractive possibility to justify Pac1-mediated repression would be to prevent mRNA accumulation, but maintain the transcription process ready for robust expression in response to external stimuli in a process conceptually similar to the RNAPII pausing described in higher eukaryotes (42). As yet, we have not been able to identify physiological conditions that prevent Pac1 activity in gene repression. Accordingly, our analysis of *mfs2* expression in stress conditions revealed that *mfs2* induction relies primarily on increased transcription initiation, while the repressive activity of Pac1 remained persistent during *mfs2* induction (Figure 5). Future studies may identify environmental conditions that allow *mfs2* and *SPBC530.02* activation by relieving Pac1-dependent repression, providing evidence supporting a ‘poised for transcriptional activation’ model. Alternatively, Pac1-dependent RNA degradation products (rather than a full-length coding mRNA) could be the functionally relevant components of the transcriptional induction of *mfs2* in stress conditions. This hypothesis, however, is currently not supported by our results as neither the full *mfs2* gene nor the Pac1 cleavage site within *mfs2* were required for survival upon oxidative stress (Supplementary Figure S7). We can also speculate that the functional importance of *mfs2* induction is the act of transcription itself, which is known to affect the chromatin structure and cause transcriptional interference (43–45). Whether such mechanisms are at play at the *mfs2* locus remains to be determined.

Given our results indicating that the accumulation of *mfs2* mRNA is not toxic to cells, another intriguing question is why are *mfs2* and *SPBC530.02* so tightly repressed during *S. pombe* vegetative growth? Indeed, the stem-loop mutations in *mfs2* that counteracts Pac1-mediated RNA cleavage did not affect growth under controlled laboratory conditions (Supplementary Figure S7). Yet, it is possible that tight control over Mfs2 transporter expression becomes critical for *S. pombe* survival in the wild, where the cells are exposed to a continuously changing environment. Indeed, *mfs2* encodes for a well-conserved protein with more than 300 predicted homologs in fungi, but intriguingly none in *S. cerevisiae* (Supplementary Figure S9A). Notably, multiple sequence alignment revealed two closely related *mfs2* orthologs in *Schizosaccharomyces* species: *S. cryophilus* (*SPOG_01195*) and *S. octospora* (*SOCG_01852*), which retained the hairpin structure and core sequence elements that we described in *S. pombe mfs2* (Supplementary Figure S9B-C). These results suggest that the *mfs2* hairpin structure appeared in a *Schizosaccharomyces* ancestor at least 120 million years ago – the estimated divergence time of *S. cryophilus*, *S. octospora*, and *S. pombe* (46). In fact, the hairpin structure could possibly be older since a more divergent *mfs2* ortholog in *S. japonicus* (*SJAG_02155*) shows the potential to form a hairpin of similar RNA secondary structure, although the hairpin sequence itself is not conserved in that species (Supplementary Figure S9B-C). Interestingly, a mammalian *mfs2* ortholog (*SPNS2*) encodes a sphingolipid transporter (47) whose disruption induces hearing loss in mice (48). Ultimately, a better understanding of *mfs2* and *SPBC530.02* gene regulation will require more knowledge about the function of the putative transporter proteins they encode.

Whereas RNAPII transcription termination at snoRNA genes depends on the Nrd1-Nab3-Sen1 (NNS) termination complex in budding yeast (49,50), it differs in fission yeast, an organism where the NNS termination pathway is not conserved. It was recently demonstrated that fission yeast snoRNA genes generally rely on the mRNA 3′ end processing machinery for transcription termination, as CPF components associate with most snoRNA genes, for which data from 3′ region extraction and deep sequencing (3′READS) often support the use of polyadenylation sites (28). However, for a subset of highly transcribed snoRNAs, no polyadenylation sites were identified, suggesting the existence of an alternative mechanism for RNAPII transcription termination. Accordingly, we show here that (i) Pac1 depletion causes readthrough transcription at *snU3*, *snU32*, and *snR88* snoRNA genes (Figure 1E and F) and (ii) that secondary structure prediction indicates potential Pac1 RNA substrates at the 3′ end of *snU3* and *snU32* snoRNAs (Supplementary Figure S2). Together, these data support a model whereby Pac1 triggers cleavage-dependent transcription termination of select snoRNAs independently from the CPF 3′ end processing machinery. In budding yeast, snoRNA transcription termination generally occurs independently of the endoribonuclease Rnt1 (51), with the exception of two snoRNAs (U3 and snR40) where Rnt1 cleaves snoRNA precursors downstream of the mature sequence, triggering transcriptional termination and 3′ end snoRNA maturation (52,53). These results highlight a diversity of RNAPII termination pathways within the snoRNA class of genes. In human cells, most snoRNAs are expressed from introns of host genes, but a small fraction of snoRNA genes are expressed as independent units, including U3, U8 and U13. How transcription terminates for those independent snoRNAs is still unclear, but as an NNS-like transcription termination pathway does not appear to be conserved in humans, it is plausible that the mechanism resembles one of those employed in *S. pombe* for snoRNA termination.

Based on our ChIP-seq data, we also found that for a large subset of Pac1-associated genes, no transcription termination defects were detected after Pac1 nuclear exclusion. At least three possibilities could explain the absence of termination defects at Pac1-bound genes: (i) the association of Pac1 with some of those genes could be spurious and not supported by any functional relationship, (ii) Pac1 could function in RNA maturation processes that occur post-transcriptionally, with no consequences on transcription termination and (iii) redundant mechanisms of transcription termination could ensure proper RNAP termination in the absence of Pac1 function. For instance, the association of Pac1 with C/D box snoRNA genes is likely to contribute to snoRNA processing. Specifically, Pac1 nuclear exclusion did not affect RNAPII density profiles, but lead to the accumulation of 5′-extended precursors at Pac1-bound C/D box snoRNAs, suggesting that Pac1 functions in snoRNA biogenesis (Supplementary Figure S3). This observation is consistent with the role of the *S. cerevisiae* Pac1 ortholog, Rnt1, in the co-transcriptional removal of the m7G cap, facilitating 5′ maturation of C/D box snoRNAs (6).

In budding yeast, Rnt1 cleavage of a stem-loop structure at the rDNA 3′ETS triggers RNAPI termination by a mechanism that relies on the 5′-3′ Rat1 exonuclease (torpedo) (13). As Pac1 is strongly recruited near the 3′ETS of the fission yeast rDNA (Figure 1B), we anticipated RNAPI termination defects in Pac1 mutants. Yet, our ChIP-qPCR assays did not reveal any changes in RNAPI occupancy upon Pac1 nuclear exclusion (Supplementary Figure S1B), suggesting that Pac1 is not required for RNAPI termination in *S. pombe*. Besides the torpedo-dependent mechanism of RNAPI transcription termination, a second ‘roadblock mechanism’ has been described in budding yeast (54,55). This mechanism involves binding of a transcription termination factor (Nsi1 in *S. cerevisiae*) to a terminator sequence element downstream of the rRNA repeat, which promotes transcription termination by pausing incoming RNAPI, likely through direct interaction with its Rpa12 subunit (56,57). The torpedo and roadblock mechanisms of RNAPI termination could be functionally redundant in fission yeast, explaining why Pac1 nuclear depletion did not result in read-through RNAPI. Future studies are needed to clarify the relative contribution and potential functional overlap between the torpedo and roadblock mechanisms in transcription termination of fission yeast RNAPI.

In summary, our study provides new insights into mechanisms of transcription termination at genes that encode structural noncoding RNAs, such as snRNAs and snoRNAs, as well as discloses further evidence for miRNA-independent roles of Drosha-related proteins in gene regulation.

## Supplementary Material

gkab654_Supplemental_Files
