# Supplementary material for: Co-transcriptional RNA cleavage by Drosha homolog Pac1 triggers transcription termination in fission yeast
Source: Nucleic Acids Res. 2021 Aug 5;49(15):8610–24. doi: 10.1093/nar/gkab654 (PMC8421224; doi:10.1093/nar/gkab654)
Supplement: gkab654_Supplemental_Files [file gkab654_supplemental_files.zip › Supplementary Figures.pdf]

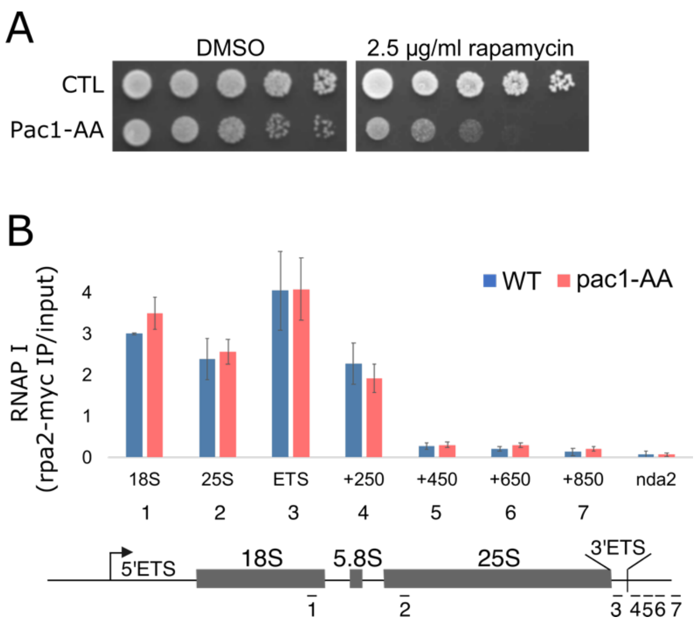

Supplementary Figure 1

A- Five-fold serial dilutions of control (CTL) and Pac1 anchor-away (Pac1-AA) strains were spotted on rapamycin-free (left) or rapamycin-containing (right) minimal media and incubated during 3 days at 30°C.

B- *Top*, ChIP-qPCR analysis of tagged RNAPI subunit (Rpa2-myc) in wild-type and Pac1-AA strains on the rDNA repeats after the addition of rapamycin at 2.5 µg/mL for 2h. *Bottom*, schematic the rDNA gene. Bars under the rDNA indicate the positions of PCR products used for RNAPI ChIP analyses. n=3 biological replicates.

A

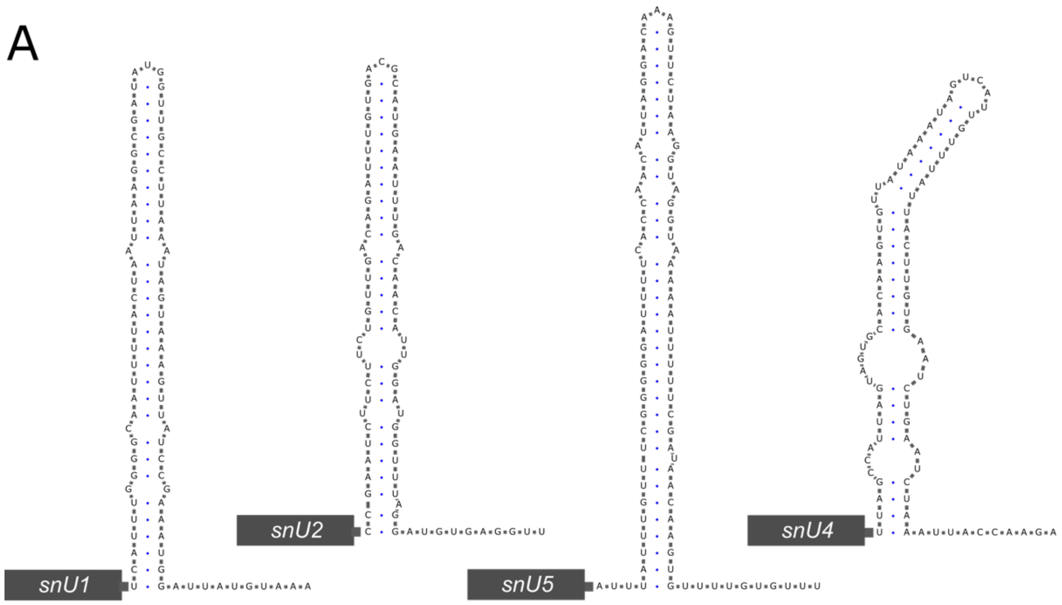

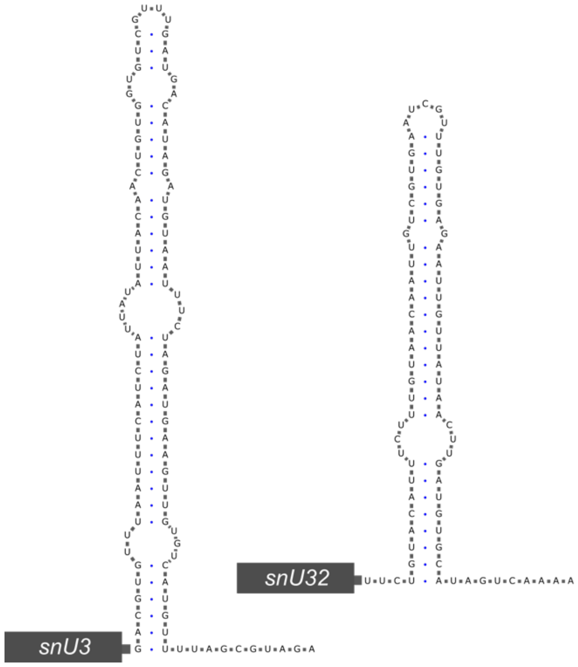

B

snoRNAs	total	Pac1-bound	% bound
**intronic**	11	0	0
**polycistronic**	9	0	0
**monocistronic**	37	15	41
|-------- C/D	17	9	53
|----- HAC/A	20	6	30
**total**	57	15	26

Supplementary Figure 2

A- Secondary structure prediction of the sequence downstream of snRNAs and selected snoRNAs for which transcriptional readthrough was observed in the absence of Pac1.

B- Distribution of total and Pac1-bound snoRNAs into intronic, polycistronic, and monocistronic groups. For the monocistronic group, snoRNAs are further divided into the HAC/A and C/D box snoRNAs classes.

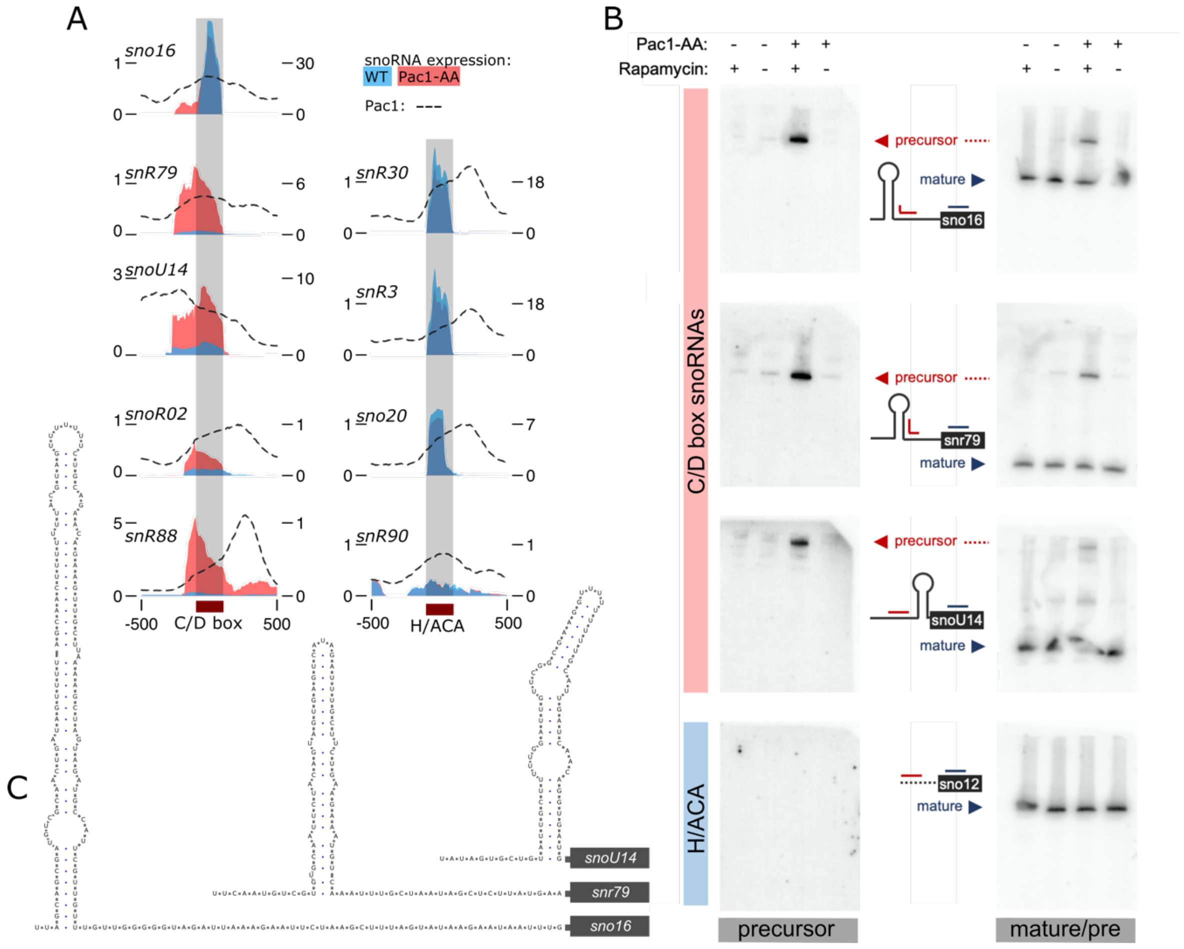

Supplementary Figure 3

A- Normalized RNA-seq read coverage over selected Pac1-bound snoRNAs in Pac1 anchor-away (Pac1-AA, in red) and control strain (in light blue) 2h after rapamycin treatment (right axis, the overlay of red and light blue is rendered as a darker shade of blue) and ChIP-seq read coverage for Pac1-TAP (left axis, dotted black line). The coverage is expressed in thousands reads mapped and averaged over two biological replicates. Pac1-bound snoRNAs that are already shown in more details in Fig. 1F (*snu3*, *snu32*) or for which no RNA-seq reads were recovered from our RNA-seq experiments (*sno12*, *snR10*, *snR61* and *snoZ16*) are not presented. snoRNAs are divided between C/D (left) and HAC/A (right) box.

B- Northern blot analysis of snoRNA precursors (red probes, left panels) or mature snoRNAs (blue probes, right panels) in control and Pac1-AA strains treated with 2.5 µg/ml rapamycin or solvent alone (DMSO) for two hours. For *sno12*, a H/ACA box snoRNA not targeted by Pac1, no precursor could be detected.

C- Predicted secondary structure of the sequence upstream of selected C/D box snoRNA for which an accumulation of precursors was observed in the absence of Pac1.

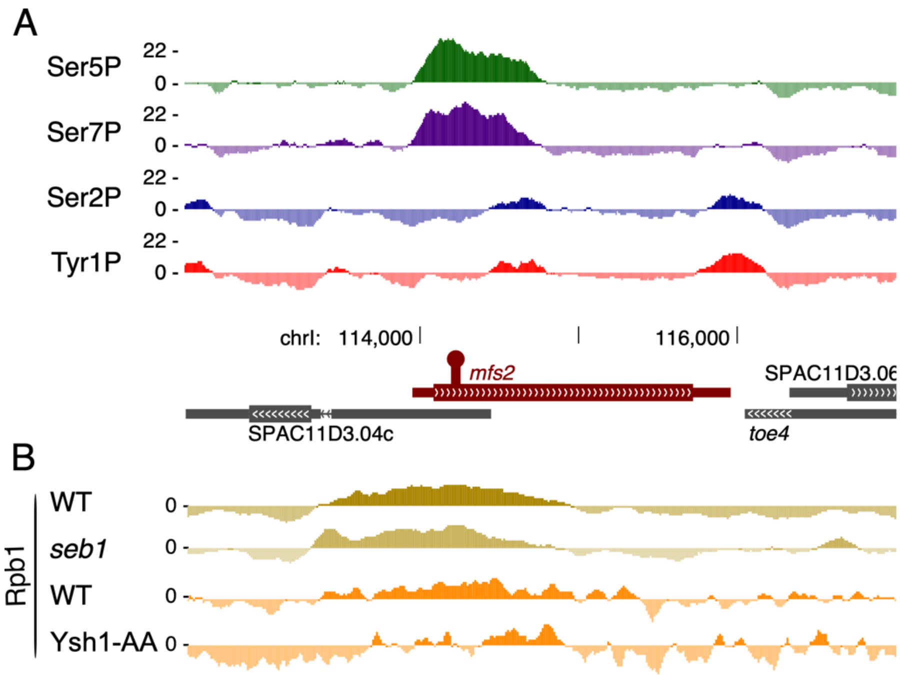

Supplementary Figure 4

A & B- Normalized ChIP-seq read coverage over the *mfs2* genes for the indicated RNAPII CTD phosphorylation marks (A) and for RNAPII subunit Rpb1 (B) in wild-type and after Seb1 depletion in a strain where *seb1* is under the control of thiamine-sensitive *nmt41* promoter, or upon rapamycin-dependent cytoplasmic relocalization of Ysh1 using an anchor-away construct (Ysh1-AA).

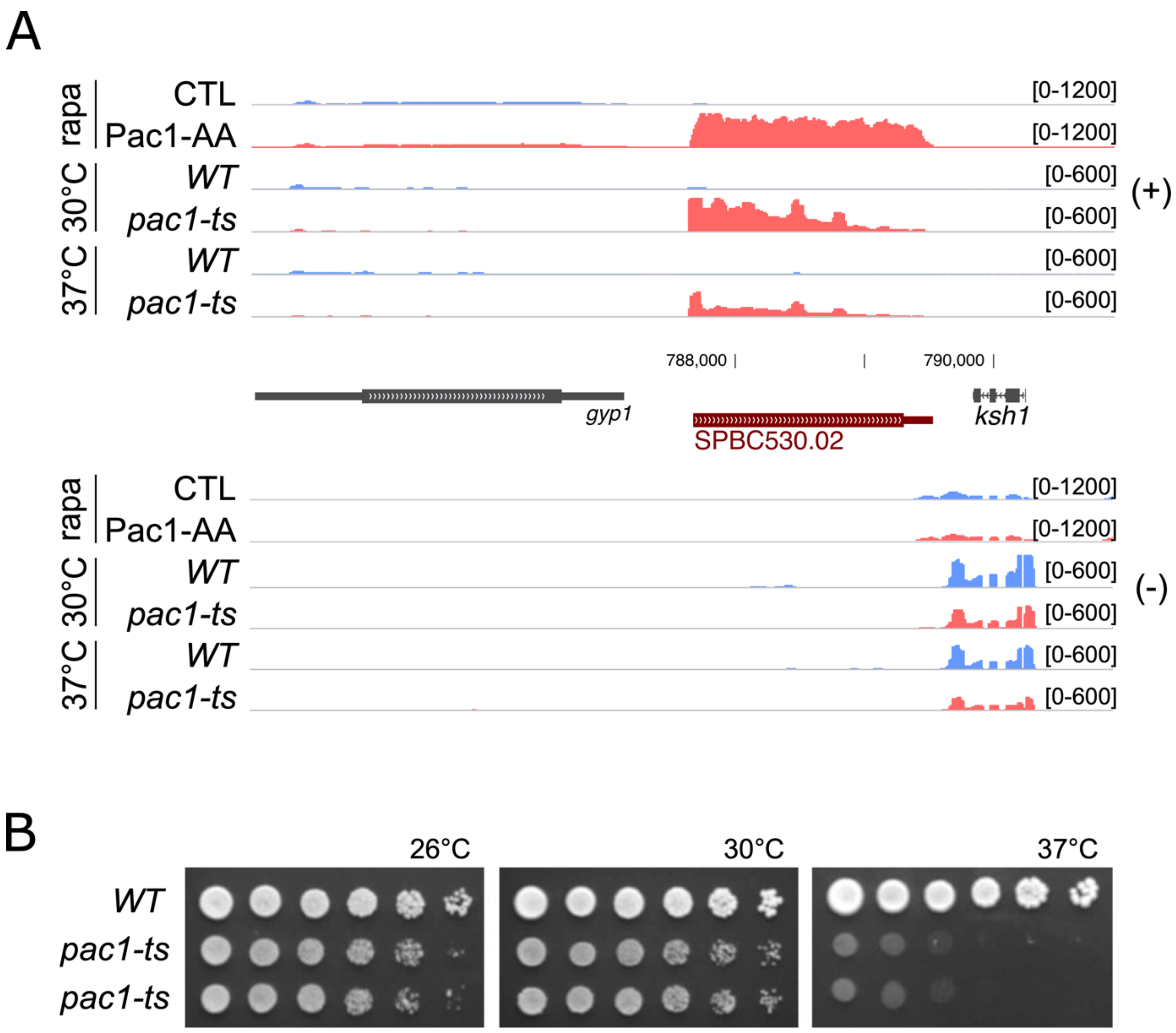

Supplementary Figure 5

A- Normalized RNA-seq read coverage averaged over two replicates centered on the *SPBC530.02* gene in control wild-type (WT) strains, upon rapamycin-dependent Pac1 nuclear depletion (Pac1-AA), and Pac1 inactivation using a thermosensitive mutant (*pac1-ts*) grown at the semi-restrictive temperature of 30°C or shifted 2h at the restrictive temperature of 37°C.

B- Five-fold serial dilutions of wild-type (WT) and two *pac1-ts* clones were spotted on YES plates and incubated during 3 days at 26°C, 30°C, and 37°C.

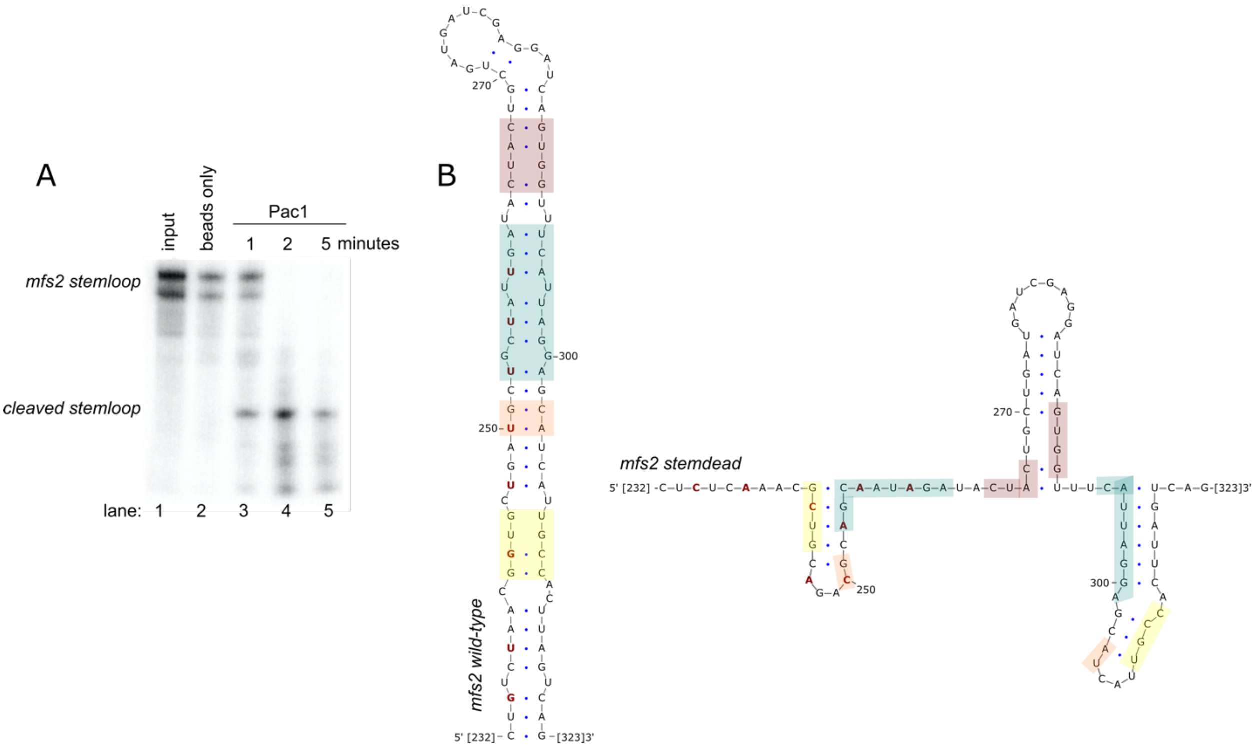

Supplementary Figure 6

A- Pac1 cleaves the *mfs2* stemloop *in vitro*. The input (lane 1) is the product of run-off transcription of the wild-type *mfs2* stemloop with 50-nt of surrounding upstream and downstream sequences. The expected size of the transcription product is about 210 nucleotides which corresponds to the upper band on the gel. As the stemloop RNA structure is digested by recombinant Pac1 on beads, cleavage products appear on the gel (lanes 3-5). To control against the activity of potential contaminating RNases, the affinity purification and cleavage assay were also performed using *E. coli* extracts that did not express Pac1: this is the *“beads only”* condition (lane 2).

B- Predicted RNA secondary structure at the beginning of the *mfs2* mRNA in wild-type and in the *mfs2 stemdead* mutant. The nucleotides mutated in the *mfs2 stemdead* mutant are highlighted in red. The indicated positions are numbered relative to the beginning of the genes. The colored boxes highlight regions of high structural and sequence homology between wild-type *mfs2* and its *SPBC530.02* paralog.

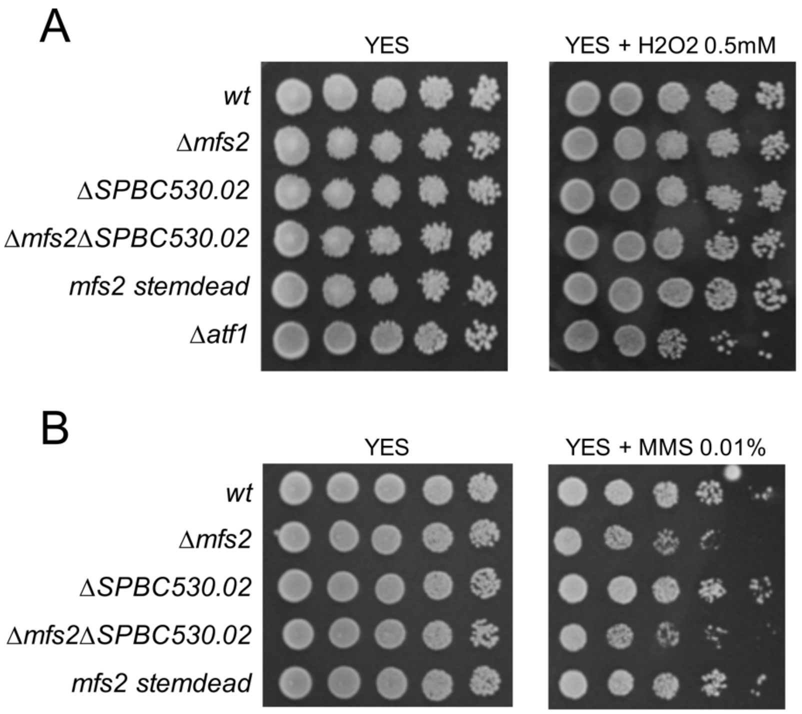

Supplementary Figure 7

A & B- Five-fold serial dilutions of the indicated strains spotted on YES plates supplemented with the 0.5 mM H_2O_2 (**A**) or 0.01% MMS (**B**) were incubated during 3 days at 30°C.

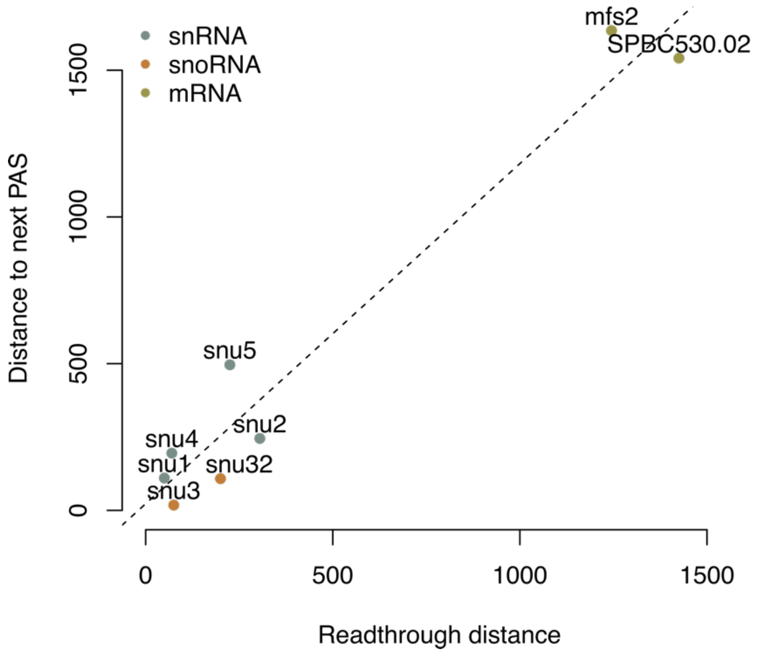

Supplementary Figure 8

Correlation between the breadth of the RNAPII termination defects observed upon Pac1 inactivation (defined as the difference of position where the RNAPII signal drops to half its maximum wild-type level between the Pac1-AA and control strains) and the distance between the Pac1 target site (stem-loop) and the next poly(A) signal consensus sequence (AAUAAA). Correlation values are 0.975 for Pearson correlation and 0.761 for Spearman correlation. A fitted linear regression is represented by a dotted black line with an adjusted R-square of 0.94 and a p-value of 3.6e-05.

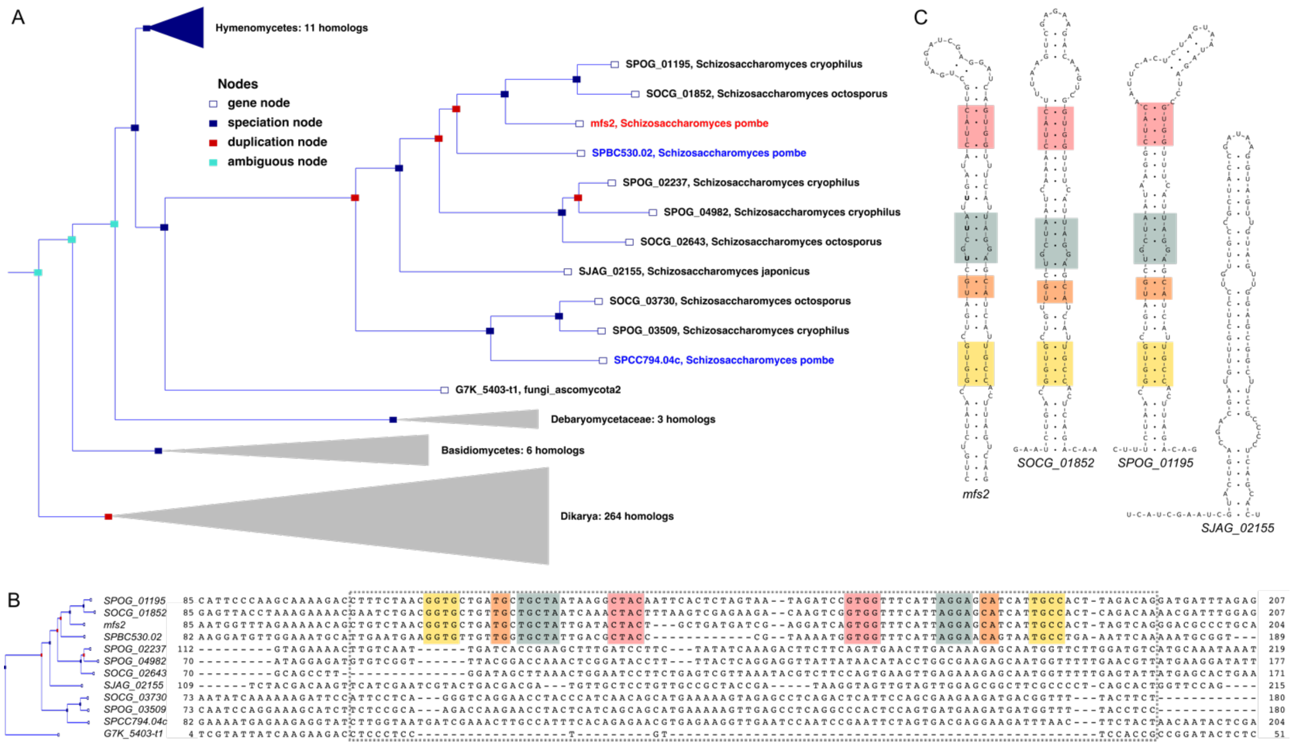

Supplementary Figure 9

A- Gene tree of the closest *mfs2* homologs (paralogs are written in blue, orthologs in black) as predicted by Fungal Compara.

B- Multiple sequence alignment of the closest *mfs2* homologs. The alignment was made globally on the complete ORFs, but only a section, bearing the *mfs2* stemloop (dotted box) is shown. The colored boxes highlight regions of high structural and sequence conservation.

C- Predicted secondary structure of the sequence of *mfs2* closest orthologs in *S. octosporus*, *S. cryophilus* and *S. japonicus*. The colored boxes highlight the same regions of high structural and sequence conservation as in Supplementary Fig. 9B.
