# Supplementary material for: Co-transcriptional RNA cleavage by Drosha homolog Pac1 triggers transcription termination in fission yeast
Source: Nucleic Acids Res. 2021 Aug 5;49(15):8610–24. doi: 10.1093/nar/gkab654 (PMC8421224; doi:10.1093/nar/gkab654)
Supplement: gkab654_Supplemental_Files [file gkab654_supplemental_files.zip › supplementary_table_caption.docx]

**Supplementary table S1:** List of genes identified as associated with Pac1 from Pac1-TAP ChIP sequencing data.

**Supplementary table S2:** Differential gene expression analysis for the full transcriptome in Pac1 defective mutants (*pac1-ts* vs wt at 30°C and 37°C and Pac1-AA vs CTL).

**Supplementary table S3:** List of *S. pombe* strains used in this study.

**Supplementary table S4:** List of oligonucleotides used in this study.

**Supplementary table S5:** List of accessions numbers for the high throughput sequencing data used in this study.
